# Zn-alloy provides a novel platform for mechanically stable bioresorbable vascular stents

**DOI:** 10.1371/journal.pone.0209111

**Published:** 2019-01-02

**Authors:** Christoph Hehrlein, Björn Schorch, Nadia Kress, Amina Arab, Constantin von zur Mühlen, Christoph Bode, Thomas Epting, Jörg Haberstroh, Lilly Mey, Hans Schwarzbach, Ralf Kinscherf, Vitus Stachniss, Stefanie Schiestel, Adalbert Kovacs, Harald Fischer, Ernst Nennig

**Affiliations:** 1 Department of Cardiology and Angiology I, Heart Center—University of Freiburg, Faculty of Medicine, University of Freiburg, Germany; 2 Department of Laboratory Medicine, Medical Center—University of Freiburg, Faculty of Medicine, University of Freiburg, Freiburg, Germany; 3 Division of Experimental Surgery, Center for Experimental Models and Transgenic Services, Medical Center—University of Freiburg, Faculty of Medicine, University of Freiburg, Freiburg, Germany; 4 Institute for Anatomy and Cell Biology, Dept. of Medical Cell Biology, Philipps-University Marburg, Marburg, Germany; 5 Department of Operative Dentistry and Endodontics, Dental School, University of Marburg and University Medical Center Giessen and Marburg, Marburg, Germany; 6 Limedion GmbH, Mannheim, Germany; 7 Optimed GmbH, Ettlingen, Germany; University of North Texas, UNITED STATES

## Abstract

Metallic Zn alloys have recently gained interest as potential candidates for developing platforms of bioresorbable vascular stents (BVS). Previous studies revealed that Mg alloys used for BVS can degrade too early, whereas PLLA materials may fail to provide effective scaffolding properties. Here we report on results of a new bioresorbable, metallic stent made from a Zn-Ag alloy studied in a porcine animal model of thrombosis and restenosis. While the tensile strength (MPa) of Zn-3Ag was higher than that of PLLA and resembled Mg’s (WE43), fracture elongation (%) of Zn-3Ag was much greater (18-fold) than the PLLA’s or Mg alloy’s (WE43). Zn-3Ag exposed to HAoSMC culture medium for 30 days revealed degradation elements consisting of Zn, O, N, C, P, and Na at a 6 nm surface depth. Platelet adhesion rates and blood biocompatibility did not differ between Zn-3Ag, PLLA, Mg (WE43), and non-resorbable Nitinol (NiTi) stent materials. Balloon-expandable Zn-3Ag alloy BVS implanted into iliofemoral arteries of 15 juvenile domestic pigs were easily visible fluoroscopically at implantation, and their bioresorption was readily detectable via X-ray over time. Histologically, arteries with Zn-3Ag BVS were completely endothelialized, covered with neointima, and were patent at 1, 3, and 6 months follow-up with no signs of stent thrombosis. Zn-3Ag alloy appears to be a promising material platform for the fabrication of a new generation of bioresorbable vascular stents.

## Introduction

Stents have become clinical routine worldwide as a standard of care to treat coronary atherosclerotic obstructions since their introduction by Jaques Puel 30 years ago [1]. However, permanent metallic vascular stent implants have specific drawbacks. Their limitations include long-term endothelial dysfunction, delayed re-endothelialization, thrombogenicity [2,3], chronic vessel wall cellular injury [4], chronic inflammatory reactions [2,3], the inability to adapt to growth in young patients or to adapt to atherosclerotic burden by positive vessel remodeling in later years, lack of physiologic vessel constriction and problematic surgical revascularization of the stented segment [2,5,6]. A clinically relevant problem of stents is in-stent restenosis, which can be effectively reduced either by intrinsic anti-proliferative characteristics [7] or anti-proliferative drug coatings [5,6]. Currently, bioresorbable endovascular stents made from medical grade PLLA and from magnesium coated with anti-proliferative agents are approved for clinical use in coronary arteries [5,6].

### Bioresorbable polymer stents

Bioresorbable stents made of polymers have been developed and investigated for many years. Initial animal experience with the biodegradable polymers polyethylene terephthalate (PET) exhibited excessive inflammation and neointima formation in porcine coronary arteries [8]. Next-generation polymer stents made of poly-*l*-lactic acid (PLLA) were introduced and demonstrated reduced inflammation, although restenosis was still observed in small diameter vessels even when anti-proliferative drug coatings were applied [6]. Overall, material disadvantages such as low collapse pressure, brittleness, hydrophilicity, acidic or gaseous degradation, as well as early elastic recoil of bioresorbable polymer platforms need to be overcome [9, 10]. Polymer stents have so far been acknowledged as having inherited inferior mechanical properties compared with non-resorbable metallic stents [11]. All bioresorbable polymer stents currently approved for clinical use are outfitted with anti-proliferative drug coatings [12].

### Bioresorbable metallic stents

Although corrosion is generally considered a drawback in metallurgy, corrodibility of certain metals consisting primarily of non-toxic trace elements could be an advantage in their application as biodegradable implants. Magnesium is a well-known bioresorbable metal involved in over 300 cellular biological reactions [2,3]. However, magnesium implants rapidly degrade and corrode in aqueous environments like body fluid, and rapid degradation of magnesium implants can result in tissue overload with degradation products [3,13]. Accelerated magnesium degradation may cause loss of mechanical integrity after a brief period of time which can compromise its scaffolding properties as a vascular implant material [14,15]. Magnesium alloyed with other elements such as aluminum, manganese, and rare earth elements decrease the degradation rates of pure magnesium at the expense of potentially toxic cellular effects [16]. Still, even the more stable magnesium alloys such as WE43 have revealed the tendency for unwanted rapid degradation leading to stent breakage, thrombosis, as well as restenosis caused partly by H_2_ production creating gas bubbles and cavities in surrounding tissues [13,17].

### Bioresorbable versus non-resorbable stents

An ideal bioresorbable stent should provide excellent X-ray visibility (e.g. high material density) and scaffolding properties preventing vessel collapse for 3 to 6 months while carrying a very low risk of material fatigue and stent fracture after expansion in atherosclerotic vascular lesions. New bioresorbable stents with improved fracture elongation, slow degradation characteristics and excellent biocompatibility need to be developed. The trace element zinc is a promising candidate for a bioresorbable stent platform [18,19]. The gold standard of non-resorbable metallic stent platforms are cobalt-chromium or Nitinol (NiTi) alloys which exhibit great mechanical stability [2, 20]. However, stent fractures due to inferior fracture elongation properties have been reported in cobalt-chromium stents [20]. We therefore sought to investigate mechanical properties and biocompatibility of a new zinc alloy (Zn-3Ag) stent material. This alloy is characterized by a high fracture elongation and ductility compared with PLLA, WE43, and non-degradable stent materials (NiTi). We further report here on our initial *in vivo* results of this bioresorbable vascular stent made from Zn-3Ag implanted into porcine iliofemoral arteries.

## Materials and methods

### Production and testing of Zn-3Ag alloy stent material

In the initial casting process, a pre-alloy was prepared by adding Zn to Ag (ratio 4:1) to reduce the melting point from 962°C to 650°C by creating a pre-alloy with the composition of Zn (80 wt-%) and Ag (20 wt-%). An Indutherm VC 600 casting machine was used for casting, putting inert gas in the melting chamber. A graphite cylinder with an outer diameter of 100 mm, inner diameter of 12 mm and 150 mm long was used as the casting mold. During casting, overpressure of 0.2 bar in the melting chamber and pressure of -0.8 bar in the casting chamber resulted in densely casted rods. The final alloy (97 wt-% Zn and 3 wt-% Ag, Zn-3Ag) was produced by melting corresponding amounts of zinc and pre-alloy during the second casting procedure. Specific compositions of pre-alloy and Zn-3Ag alloy were measured via Atom Absorption Spectroscopy (AAS). Extrusion was performed by heating a 10 mm diameter Zn-3Ag rod to 300°C and then pressing it through a hardened steel mold (AISI/SAE 1.2361) having an 3 mm bore using a 30 ton hydraulic press. We measured the mechanical characteristics (e.g., yield strength, tensile strength, and fracture elongation) of the novel alloy according to international quality standards (DIN EN ISO 6892–1).

### X-Ray photoelectron spectroscopy (XPS) analysis of surface elements

Surfaces changes in the material samples immersed in HAoSMC cell culture medium were subjected to X-Ray Photoelectron Spectroscopy (XPS) using a Thermo Fisher Scientific XP spectrometer. The pressure inside the UHV-chamber was below 10^−9^ mbar during measurements. Spectra were taken from 0 to 1100 eV using Mg Kα radiation (1254 eV). In the case of WE43, 0 to 1400 eV spectra using Al Kα radiation (1487 eV) were applied with a pass energy of 75 eV, dwell time of 20 ms, and a step size of 1 eV. XPS data were taken before and after sputtering with argon ions for 60 s, 180 s, and 300 s. The argon ion energy was 2 keV and a current of 3 μA was applied onto a surface area of 25 mm^2^, resulting in an estimated sputter rate of 6 nm/min. As a result, the recorded spectra corresponded to a depth of 6 nm (60 s sputtering), 18 nm (180 s sputtering), and 30 nm (300 s sputtering). Data were analyzed using the software Avantage (Thermo Fisher Scientific XP).

### Atom absorption spectroscopy (AAS) analysis of Zn release

Biodegradation was induced by immersing Zn-3Ag samples in simulated body fluid (SBF), exchanging the fluid after 5, 8, 12, 20, 22, 27, 32, 45, 55, and 85 days. Any deposits left in the fluid were dissolved with nitric acid. Zn concentrations were measured by Atom Absorption Spectroscopy (AAS) at each time point. SBF contained 137 mM NaCl, 4.2 mM NaHCO_3_, 3 mM KCl, 1 mM K_2_HPO_4_, 1.5 mM MgCl_2_, 1.9 mM CaCl_2_, 0.5 mM Na_2_SO_4_, 50 mM Tris, and 30 mM HCl at pH 7.4. Material discs of 15 mm diameter and 3 mm thickness yielding a surface area of 4.9 cm^2^ were used with a sample-to-solution ratio of 1 cm^2^/20 mL. Before submersion, material samples were polished using SiC abrasive discs of 1/1000 grit size and then cleaned with 2-propanol. The immersing procedure took place at 37°C in a climate chamber, and solutions were stirred continuously. The materials degradation rate was calculated as follows: $rate\;\left[\frac{\mu m}{year}\right]=rate\;\left[\frac{mg}{{cm}^{2}*day}\right]*\frac{365\;\left[days\right]*[{cm}^{2}*\mu m]}{\left[year]*density[mg\right]}$ with a density of 7,3 g/cm^3^ (0,73 mg/cm^2^*μm) of Zn-3Ag.

### Samples, sample extract preparation and manufacturing of Zn-3Ag vascular stents

Pure Zn, Zn-3Ag, WE43, and Nitinol were prepared as round, electropolished discs from metal tubes, and PLLA medical grade discs were cut from PLLA tubes. All discs were sized to 3 mm diameter and a thickness of 0.2 mm and sterilized with ethylene oxide. Material extracts for cytotoxicity tests were prepared as follows: 1 mL of appropriate cell culture medium together with one material disc was incubated for 24 h at 37°C under steady agitation. The discs were removed and the eluates stored at 4°C in the dark for up to 14 days. Zn-3Ag balloon-expandable stents were produced as electropolished, standard open-cell stents at a length of 20 mm with a mean strut thickness of 180 μm to be deployed via balloon expansion to an external diameter of 6 mm.

### Viability of human aortic smooth muscle cells (HAoSMC) in sample contact

Human aortic smooth muscle cells (HAoSMC, PromoCell, Heidelberg, Germany) were grown in culture flaks using SMC medium 2 (PromoCell) to 80% confluency at 37°C in humidified air containing 5% CO_2_. Passaging was performed with the detachment kit (PromoCell) at a ratio of 1:4. All HAoSMCs were studied between the fifth and ninth passage. 5x10^4^ cells per well in 1 mL medium were grown for 24 h before further tests. A cell proliferation kit (CFSE, PromoCell) was used to measure proliferation of HAoSMCs. Warmed sterile Dulbecco´s PBS *(PAA Cell Culture Company*, *Cambridge*, *USA)* mixed with 5 μM CFSE was added to 5x10^4^ HAoSMC/mL. Cell suspensions were added on cover slip glass in 24-well plates with or without a material disc positioned in the center of the wells. Cells on the surface of the material discs (surface contact) and cells periphery of the 24-well plates not in contact with the discs (no contact) were fixed with 4% paraformaldehyde (PFA) in PBS. HAoSMC apoptosis was determined using propidium iodide solution (BD Biosciences, USA) with staurosporine (PromoCell) used as positive control. HAoSMC nuclei were stained with DAPI mounting medium (Vectashield, Vector, USA). Proliferating cells in three identically-sized areas of the center (disc surface) and in three areas of the periphery of the 24-well plates (no contact) were counted using fluorescence microscopy (AxioVision).

### *In vitro* hemolysis test of material samples

Human whole blood was collected via peripheral venous puncture of a healthy donor. The blood sample was filled in an EDTA collection tube (EDTA Monovette, Sarstedt, Nümbrecht, Germany) and gently mixed to prevent clotting. Aliquots were incubated at 37°C on a roller shaker with or without a material sample disc. 1% sodium dodecyl sulfate (SDS) was used as positive hemolysis control. Following 1, 2, and 3 hours of incubation, plasma samples were obtained by centrifugation and removing cellular components. The plasma hemoglobin concentration was measured by a routine procedure in the clinical chemistry laboratory of the University Hospital Freiburg.

### *In vitro* adhesion of human platelets to material surfaces

Use of human blood samples was reviewed and approved by the Ethics Committee of the University Medical Center Freiburg, Germany (#23/15). All donors were adult and gave written informed consent at the Blood Donation Center of the University of Freiburg Medical Centre. Human thrombocytes (platelets) were obtained from fresh human buffy coats and labeled with CFSE. The CFSE-labeled thrombocytes were incubated with material discs for 3 h at 37°C under gentle agitation at a concentration of 5x10^7^ platelets per ml. As positive control, a glass slide was coated with 0.5 U of thrombin (Vascular Solutions, Minneapolis, USA). Following incubation, material discs were washed once with Tyrode’s solution and adherent thrombocytes were fixed with 4% phosphate buffered paraformaldehyde for 10 minutes. Discs were washed twice again with PBS and mounted on microscopic slides. Adherent thrombocytes (platelets) per mm^2^ were counted immediately via fluorescence microscopy (*AxioVision*).

### Porcine study protocol

Animal experiments were approved by the by the Ethics Committee of the Federal Ministry for Nature, Environment, and Consumer Protection of the state of Baden-Württemberg, Germany (G15/153). Experiments were performed on juvenile domestic swine (32–40 kg) of equal sex distribution according to the standard of care outlined in accordance with the ARRIVE guidelines of animal research [21]. Pigs were obtained from pig breeder Andreas Rein, Viehweg 1, 79206 Breisach, Germany. Each animal underwent dual antiplatelet therapy applying ASS and clopidogrel one day before the procedure and continued until termination. Swine were starved for 12 h and premedicated i.m. with 0.5 mg kg^−1^ midazolam and 20 mg kg^−1^ ketamine hydrochloride. Anesthesia was induced i.v. with 2–4 mg kg^−1^ propofol and maintained by inhalation of 1.5–2.5% isoflurane and 4 μg kg^−1^ h^−1^ fentanyl citrate. Muscle relaxation was maintained i.v. by 0.2 mg kg^−1^ h^−1^ vecuronium. After tracheal intubation, the lungs were ventilated in the volume-controlled mode at a respiratory rate of 10–15 breathes per minute, a tidal volume of 8 ml kg^−1^, and PEEP of 8 cm H_2_O. Inspired oxygen fraction (FiO2) was maintained at a rate of 0.3. Ringer's solution was infused at 10 ml kg^−1^ h^−^1. Carotid artery access was obtained via surgical cut-down. Before catheterizing the arterial system, heparin (5–10.000 IU) was administered to maintain activated clotting time >250 s. A total of 15 iliofemoral arteries in 15 juvenile domestic swine received Zn-3Ag stents and were divided into 1-month (n = 6), 3-month (n = 6), and 6-month (n = 3) follow-up study groups. At follow up animals were finally sacrificed in deep narcosis by rapid injection of 40 mmol KCl.

### Angiography after implantation of balloon expandable Zn-3Ag bioresorbable stents in porcine iliofemoral arteries

Angiograms of the iliofemoral region were obtained under fluoroscopy control pre- and post-stent implantation as well as at the 1, 3, and 6 month follow-up periods after stenting.

### Histopathology of vascular healing after implantation of Zn-3Ag bioresorbable stents in porcine iliofemoral arteries

Stented arterial samples were dehydrated in acetone and embedded in hydroxyethyl-methacrylate (Technovit 8100, Kulzer). After polymerization, the stents were cut using a rotary diamond saw (Leica SP 1600, Germany) into cross-sections of 300 μm thickness, ground and polished into 40–60 μm-thick sections using a disc sander (Exakt 400CS, Norderstedt). To easily detect the internal- and external elastic membranes, cross-sections were stained with Giemsa or toluidine blue. Two histopathologists independently analyzed the specimens by the four-eye principle with respect to circumferential neointimal strut coverage as a sign of arterial endothelialization and vascular healing after stenting.

### Statistics

Statistical analysis was performed with IBM SPSS Statistics 22 and graphic data representations were generated by Microsoft Excel. Significance for the comparison of two groups was calculated via the unpaired Students *t*-test. Multiple groups were analyzed by a multivariate ANOVA with a following pairwise comparison using the Holm-Sidak method. If not stated otherwise, graphs display average values from at least three experiments with error bars representing the standard error of the means (SEM). Significance levels were illustrated as follows: *: p < .05; **: p < .01; ***: p < .005.

## Results

### Zn-3Ag extrusion and material properties

**Fig 1:**
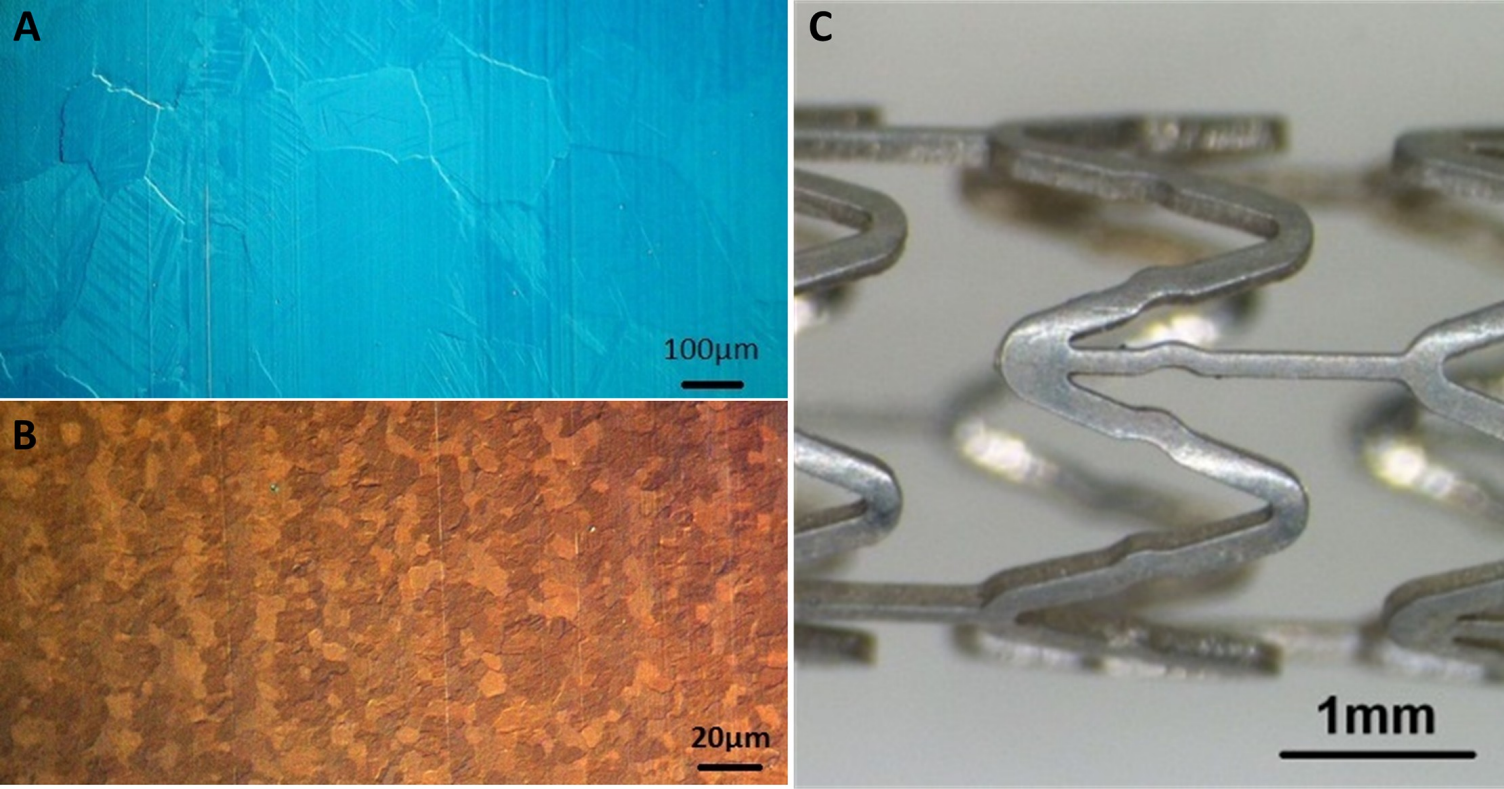
Zn-3Ag alloy used for the production of a vascular stent. Microphotographs of the grain size of Zn-3Ag alloy directly after casting **(A)** and extrusion **(B)**. Zn-3Ag stent expandable to a diameter of 6 mm is shown **(C)**.

Casted rods of Zn-3Ag exhibited grain sizes initially of over 200 μm, which were further reduced to small grain sizes by extrusion. The resulting rods had grain sizes of 5 to 15 μm (Fig 1). 20 mm long Zn-3Ag stents were laser-cut from Zn-3Ag tubes, mechanically crimped onto 6x20 mm balloons, and were expanded up to a diameter of 6 mm for surface evaluation (Fig 1). Zn-3Ag material demonstrated higher tensile strength compared to PLLA and much higher fracture elongation rates than PLLA, WE43, or pure Zn (Table 1). The mechanical stress-strain curve of Zn-3Ag is provided in S1 Fig. Mechanical curves were also taken from tubes (3 mm OD, 0,2 mm wall thickness) of Zn-3Ag and these showed lower mechanical stability as compared to the bulk material (elongation: 175%; tensile strength: 155 MPa; yield strength: 70 MPa).

**Table 1:** Mechanical properties of Zn-3Ag alloy in comparison to bioresorbable (WE43, PLLA) and non-resorbable stent materials (CoCr, NiTi).

	E-modul (GPa)	Yield strength (MPa)	Tensile strength (MPa)	Elongation (%)	Density (g/cm^3^)
CoCr (L-605)	210^[^^20^^]^	448–648^[^^20^^]^	951–1220^[^^20^^]^	3^[^^20^^]^	9.2^[^^20^^]^
NiTi (Nitinol)	83^[^^2^^]^	195–690^[^^2^^]^	895^[^^2^^]^	56^[^^2^^]^	6.7^[^^2^^]^
Mg (WE43)	44^[^^2^^]^	195^[^^3^^]^	280^[^^3^^]^	2^[^^3^^]^	1.8^[^^2^^]^
PLLA	4^[^^11^^,^^29^^]^	47–77^[^^11^^,^^29^^]^	53^[^^11^^,^^29^^]^	6^[^^11^^,^^29^^]^	1.2^[^^11^^,^^29^^]^
Zn-3Ag	95^‡^	130–145^‡^	240–260^‡^	70–135^‡^	7.2^#^

^‡^measured at Staatliche Materialprüfungsanstalt Darmstadt, Germany, according to DIN EN ISO 6892–1

^#^calculated.

### Elemental composition of different stent material surfaces assessed by XPS

**Fig 2:**
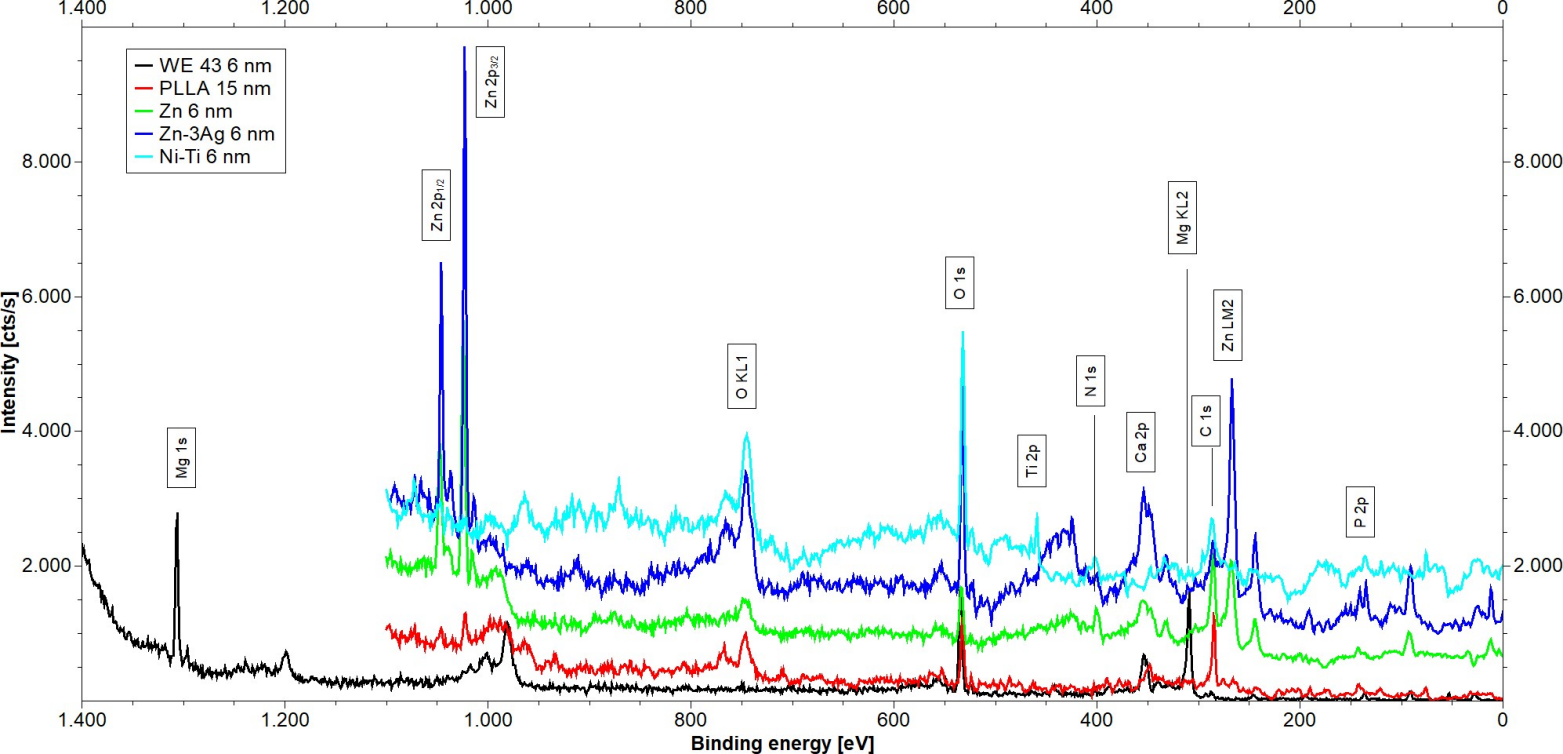
Surface composition of Zn-3Ag. Shown are XPS spectra of samples obtained after 60 s of sputtering of WE43 (black), Nitinol (red), Zn (green), Zn-3Ag (blue), and PLLA (cyan). For visibility curves were stacked by the following addition: Zn 500 cts/s, Zn-3Ag 1 000 cts/s, and NiTi 1 500 cts/s.

Only a low metal concentration (<5 at-%) and high concentrations of carbon and oxygen were identified on the surface of Zn, Zn-3Ag, and non-degradable Nitinol (NiTi) samples before sputtering. We observed a high metal concentration of 20 at-% on the WE43 surface before sputtering. Fig 2 illustrates the XP spectra of Zn, Zn-3Ag, WE43, and Nitinol samples after 60 s sputtering (6 nm).

Carbon and oxygen were detected on all sample surfaces. The surfaces of Nitinol, Zn, Zn-3Ag and PLLA also revealed nitrogen. We detected calcium on WE43 and PLLA, and phosphorus on the surfaces of Nitinol, WE43, and Zn-3Ag. We noted a low concentration of sodium on Nitinol and Zn-3Ag surfaces. With its higher carbon content, pure Zn differed from the other stent samples. The high carbon concentration and a peak position of C 1s indicated an organic film on the surface. PLLA samples showed specific incorporations of nitrogen, resulting in a change in surface composition from (C_3_O_2_)_n_ to (C_16_O_6_N)_n_. The WE43 surface composition of MgO_2.5_Ca_0.2_P_0.48_ indicated that an inorganic coating had formed on the Mg alloy. The binding energies of phosphorus signals were shifted to higher energies (135 eV), suggesting the presence of phosphite or phosphate on the surface. Elemental Ti, O, N, and C were detected in a ratio of TiO_7.3_N_1.1_C_2.2_ after sputtering the Nitinol samples. Carbon, oxygen, and calcium were observed on the PLLA surface at a 17:6:1 ratio. No silver was detected on the Zn-3Ag alloy surface even after sputtering. XPS analysis results are summarized in Fig 1 and S1 Table. The last column on the right side of S1 Table illustrates a stoichiometric comparison, calculated from the atomic composition of the different samples’ surfaces.

### AAS analysis of Zn-3Ag degradation *in vitro*

**Fig 3:**
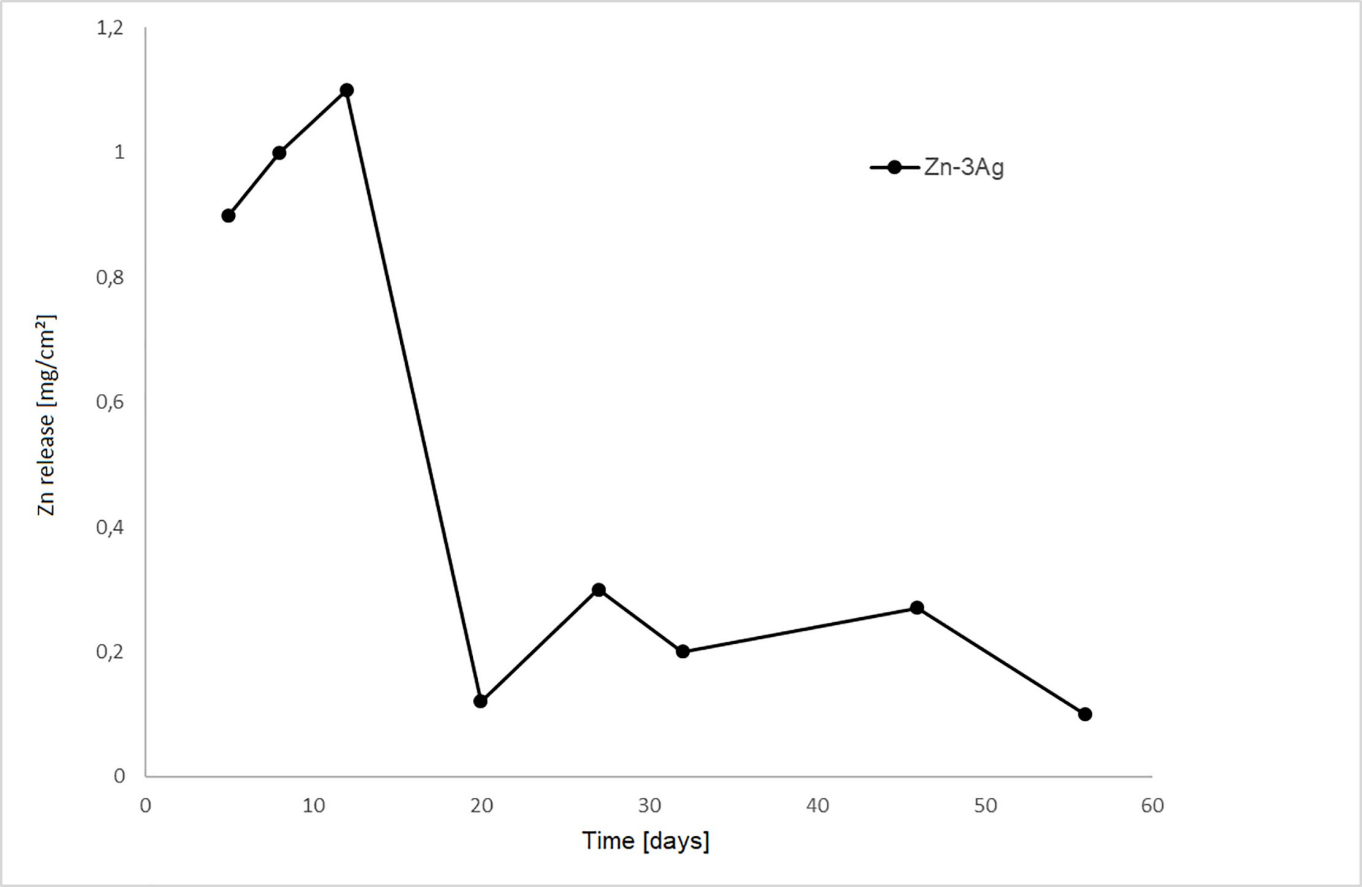
Zn-3Ag biodegradation by simulated body fluid. Zn concentration measured by atom absorption spectroscopy (AAS) after immersion of Zn-3Ag alloy samples in simulated body fluid (SBF) at the time of fluid change for 85 days were shown.

The Zn amount dissolved in the test fluid rose relatively quickly from the material surface, followed by saturation, suggesting surface coverage with corrosion products decelerating further corrosion (Fig 3). Zn-3Ag alloy corrosion equaled 0.16 mm/y. No H_2_ generation or gas bubbles were observed during Zn-3Ag degradation.

### Proliferative and apoptotic signs of human aortic smooth muscle cells (HAoSMC) in contact with different stent materials *in vitro*

**Fig 4:**
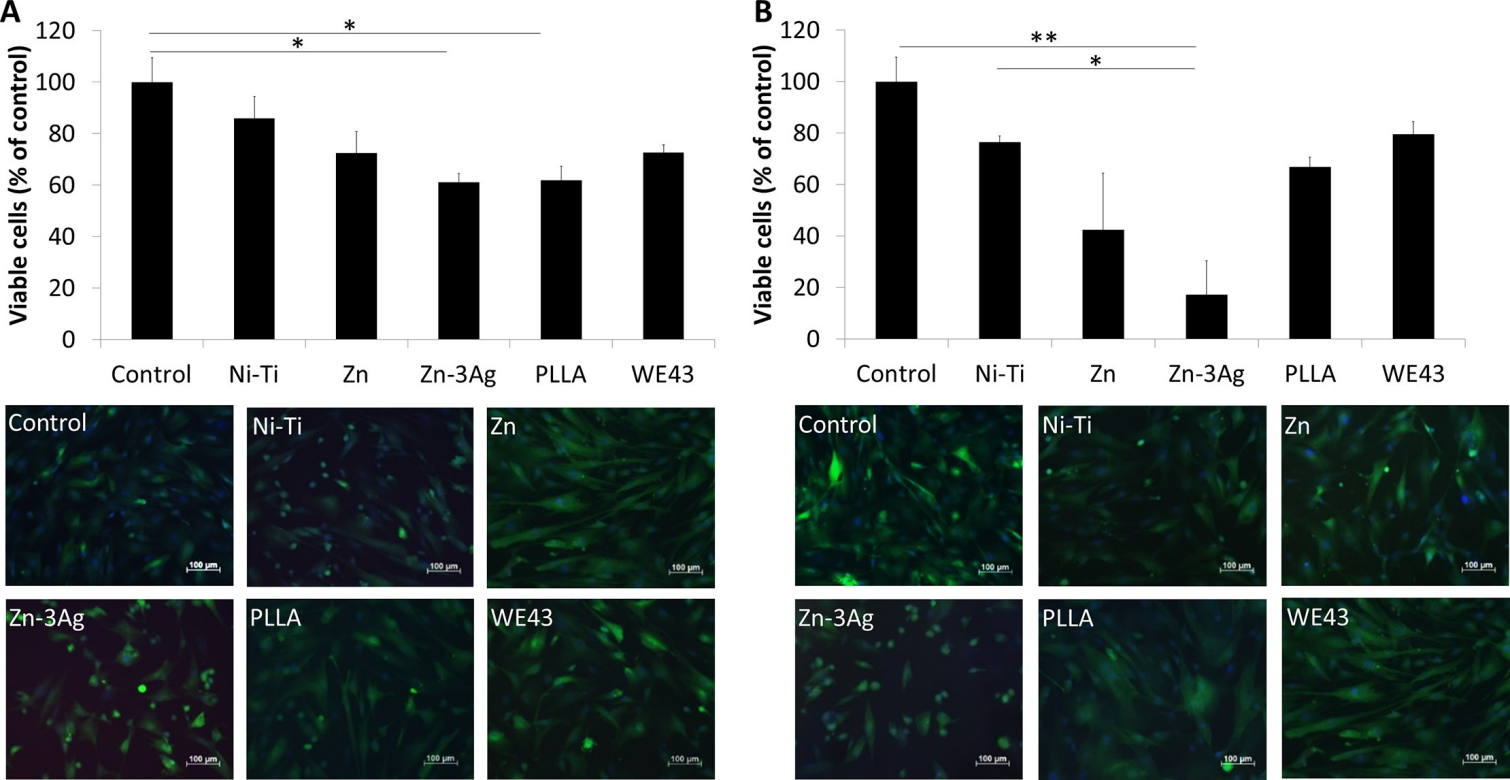
Zn-3Ag affects proliferation of HAoSMCs. Zn and Zn-3Ag reduce proliferation of human aortic smooth muscle cells (HAoSMC). Viable cells surrounding material discs **(A)**, and cells attached directly to the material **(B)** stained with a green fluorescent cell proliferation (CSFE) kit and red fluorescent propidium-iodide were microphotographed and cell numbers calculated. Significance levels are illustrated as follows: *: p < .05; **: p < .01.

The numbers of proliferating CFSE positive and metabolically active HAoSMC were found to be mild to moderately decreased in direct contact with Zn or Zn-3Ag alloy discs or eluates, but excessive cell death by apoptosis or necrosis was not observed. Findings were comparable to HAoSMC studied in wells after contact with Nitinol, PLLA, and WE43 discs (Fig 4).

### *In vitro* human whole blood hemolysis studies and platelet adhesion

**Fig 5:**
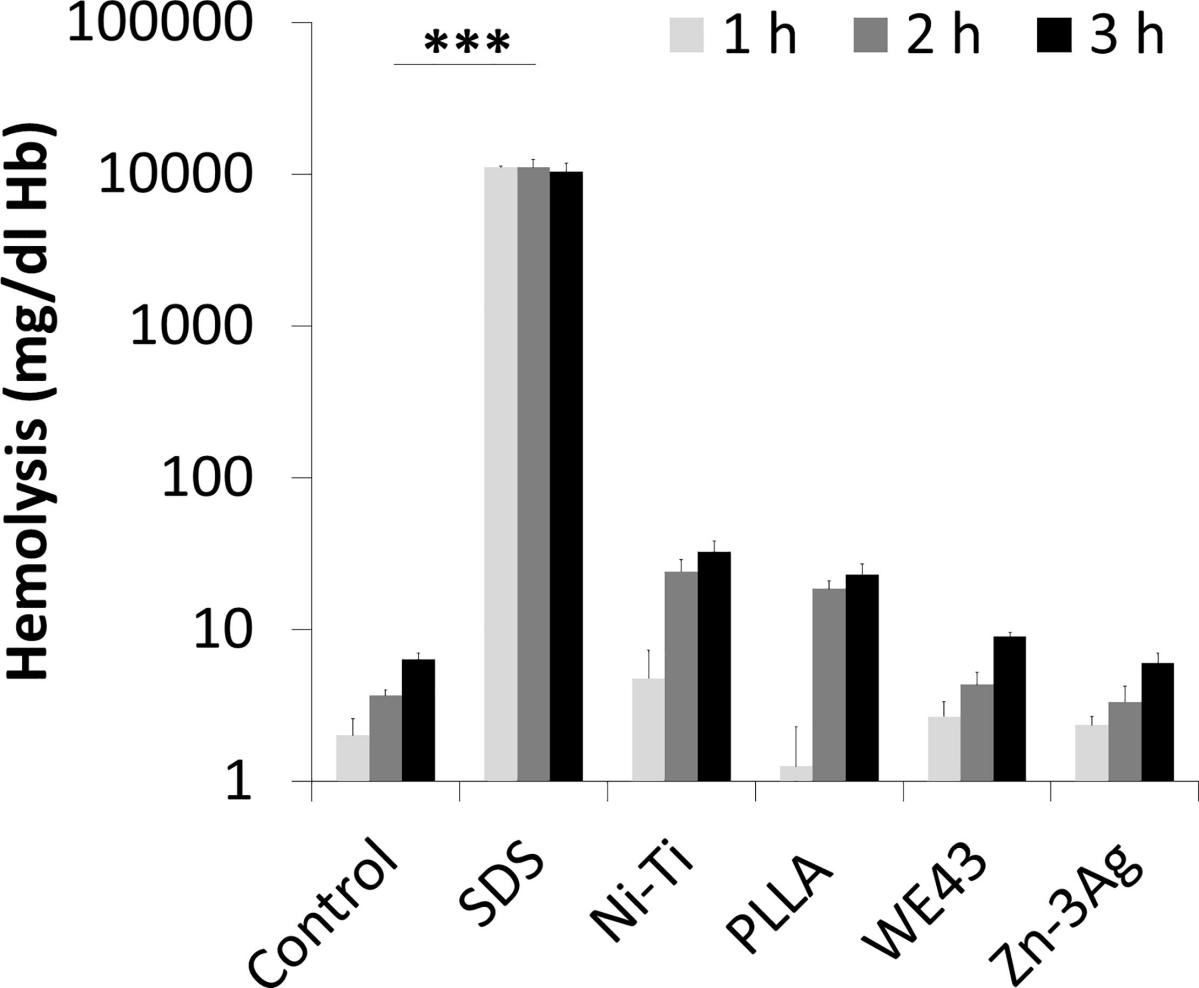
Hemocompatibility of Zn-3Ag. Human blood hemolysis in contact with the stent materials Zn-3Ag, Nitinol, PLLA, and WE43 was measured *in vitro*. Shown are the results of 6 individual experiments.

**Fig 6:**
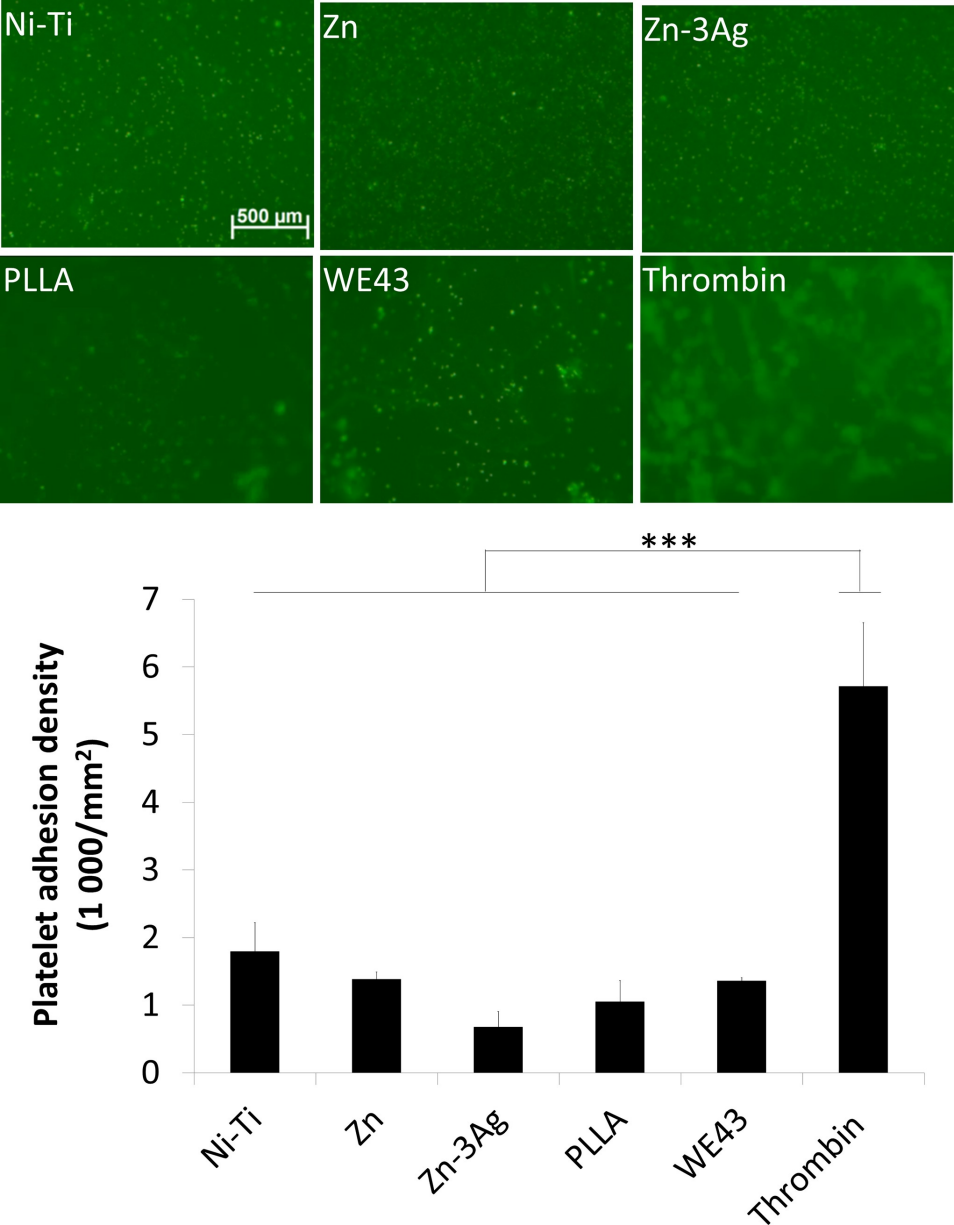
Comparable platelet adhesion on stent materials. Platelet adhesion on Zn, Zn-3Ag, Nitinol, PLLA, and WE43 surfaces was measured and quantified *in vitro*. Quantified are three independent experiments. Significance levels are illustrated as follows: *: p < .05; **: p < .01.

Material discs incubated under steady agitation with fresh EDTA anti-coagulated human blood demonstrated similar low rates of erythrocyte disruption among all study materials after 1, 2, and 3 hours of contact time by measuring the free hemoglobin content in plasma. Zn-3Ag alloy showed a hemolysis rate of 6 mg/dl hemoglobin release in 3 hours which is not significantly different from control blood samples and the stent materials Nitinol and WE43 (Fig 5). In comparison to PLLA (23 mg/dl hemoglobin/3 h, p = 0.004) the hemolysis rate of Zn-3Ag is significantly reduced,

The numbers of CFSE-labeled human thrombocytes did not differ after 3 h of incubation on all the tested surfaces of Nitinol (Ni-Ti), Zn, Zn-3Ag, PLLA, and WE43 material discs, whereas thrombin-coated positive control slides elicited massive adherence of thrombocytes (platelets) to its surfaces (Fig 6).

### Fluoroscopic and angiographic results after implanting Zn-3Ag stents in porcine iliofemoral arteries

**Fig 7:**
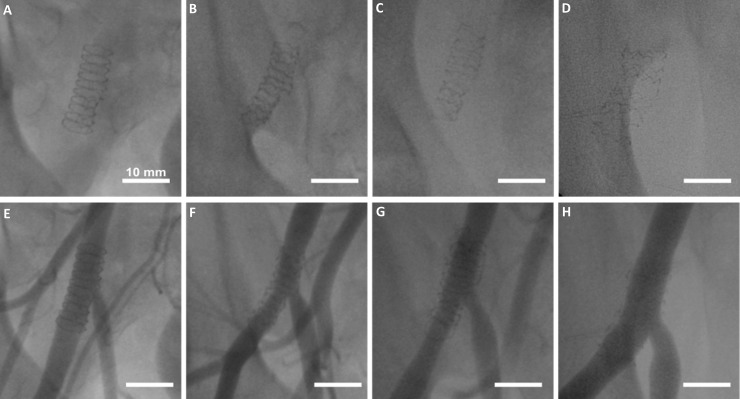
Zn-3Ag bioresorbable stents implanted in iliofemoral arteries of pigs. Fluoroscopic X-ray images of Zn-3Ag bioresorbable vascular stent in porcine iliofemoral arteries immediately post-implantation **(A)**, after 1 month **(B)**, 3 months **(C)**, and 6 months **(D)** show loss of radiopacity of the stents over time. Angiograms of Zn-3Ag stents reveal vessel patency without marked lumen narrowing or stent thrombosis post-implantation **(E)**, after 1 month (**F**) after 3 months **(G)** and after 6 months (**H**).

Fluoroscopy images of porcine iliofemoral arteries prior to the angiograms were taken immediately after Zn-3Ag stent implantation, and exhibited the stent’s complete expansion as well as clear visibility of the stent dimensions due to high radiopacity. The iliofemoral arteries’ angiograms revealed no signs of early thrombosis or post-interventional vessel failure, and only minor luminal narrowing at the 1, 3, and 6 month follow-ups (Fig 7).

### Histological evaluation of Zn-3Ag stents in porcine iliofemoral arteries

**Fig 8:**
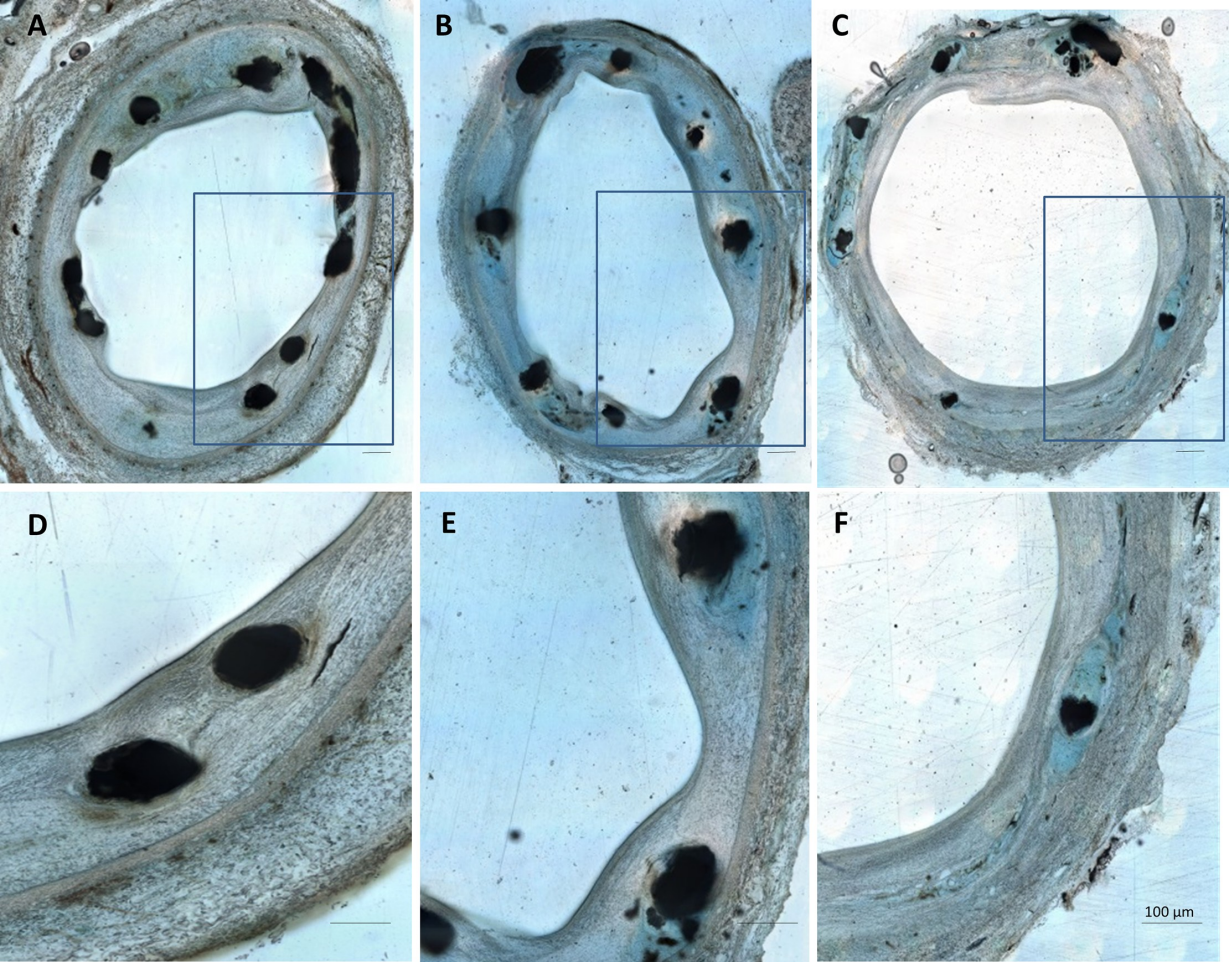
Zn-3Ag bioresorbable stents are rapidly covered by endothelium. Histologic images of representative cross-sections of porcine iliofemoral arteries stented with a Zn-3Ag bioresorbable vascular stent after 1 month **(A** and **D)**, 3 months **(B** and **E),** and 6 months **(C** and **F). D, E,** and **F** show magnified stent struts covered by neointima within the corresponding arterial cross-sections **A**, **B,** and **C**.

The histopathology of arterial cross-sections after Zn-3Ag stent implantation demonstrated circumferential coverage of the struts with neointima, indicating early vascular healing after 1 month, which continued through the 6 month follow-up period (Fig 8). We did not detect a single uncovered stent strut, indicating complete endothelialization and thus minimal risk of in stent thrombosis (Fig 8).

## Discussion

A recent clinical study in coronary arteries comparing the long-term results of bioresorbable PLLA stents with non-degradable metallic stents revealed the inferiority of PLLA stents versus metallic non-degradable stent platforms, causing more late lumen loss due to restenosis in the treated arteries [6]. Zinc (Zn) is an essential trace element participating in the production of numerous proteins and enzymes in the human body, and dietary guidelines recommend a daily oral zinc intake of 15 mg/day in male, 5–15 mg/day in female adults [22,23]. In the search for novel bioresorbable stent platforms with improved mechanical strength and scaffolding properties compared with PLLA- or magnesium alloy platforms, Zn has been suggested as a potentially promising candidate for bioresorbable stents or scaffolds [18,19]. Wires made of pure zinc were implanted into the rat aorta and endothelialized [19]. Mg alloys incorporating small amounts of Zn, such as Mg-Zn-Se alloy [24] or Zn alloys such as Zn-Mg [25] and Zn-Cu alloys [26] have been shown to degrade at a much slower rate than most other magnesium(Mg)-alloys including WE43. We found that Zn alloyed with 3 wt-% Ag is characterized by a substantially enhanced ductility over PLLA and magnesium (Table 1). The mechanical behavior of Zn-3Ag alloy in terms of fracture elongation compares favorably with either Mg-alloy [11,27] or PLLA [10,11]. The moderate degradation rate of Zn-3Ag alloy positioned between Mg and PLLA further supports its potential suitability for sufficient vessel scaffolding during the first 3–6 months of stent implantation.

Biocorrosion tests in this study revealed a slow rate of Zn ions release, corresponding to a material loss of 0.16 mm/y, for the Zn-3Ag alloy. Zn-1Mg alloy’s degradation rate was reported to be comparable at values of 0.12–0.28 mm/y [25]. Furthermore, Zn-3Ag alloy did not induce H_2_-production and gas bubbles during its degradation. We found that a Zn-3Ag stent platform elicits of mild to moderate HAoSMC cytotoxicity in direct probe contact and less HAoSMC viability than non-degradable Nitinol or bioresorbable PLLA materials. However, the rate of apoptotic cell death of HAoSMC as indicated by propidium iodide uptake was similar across all study groups. We detected no excessive apoptotic or necrotic cell death at and around Zn-3Ag stent samples, thus the anti-proliferative effects of released zinc ions may primarily be an induction of growth arrest. Our cell culture findings are consistent with previous reports of reduced HAoSMC viability caused by Zn ions [18]. Based on the proposed biphasic cellular response to zinc ions [28] we assume a low local zinc ion concentration (< 80 μM) due to the low degradation rate. Although not investigated here, these concentrations should not damage endothelial cells [29].

Our animal data showed that bare Zn-3Ag BVS demonstrate evidence of unaltered vascular healing because stent struts were completely covered by neointima already after a 4-week follow-up. Visibility and positioning of Zn-3Ag stents under fluoroscopic control were excellent. The x-ray visibility of the novel bioresorbable stent diminished slowly over time, but a stent frame remained detectable 6 months after implantation into porcine iliac arteries. Angiographic and histologic data revealed that degradation of Zn-3Ag stents in porcine iliac arteries took place without stent thrombosis or vascular occlusion, and maintained vascular scaffolding for a minimum time period of 6 months. Because clinical studies of PLLA scaffolds in patients with peripheral arterial disease and stenotic femoral arteries have not shown good results due to the scaffolds’ poor mechanical properties applying modern PLLA materials [30], the search for optimal bioresorbable vascular stents continues [31]. Zn-implants exhibited excellent biocompatibility in a rabbit model of aortic stenting [32] and as MMC bone implants [33,34]. Studies of Zn-Ag alloys have reported high fracture elongation and tensile strength data similar to our findings [35]. Lower tensile strength of our Zn-3Ag material resulted in slightly higher fracture elongation compared with previous data obtained for Zn-Ag alloys ranging from 2.5 to 7 wt-% [35]. The processes from the bulk materials to mini-tube—like deep drilling and tube drawing—greatly affect the mechanical properties of final mini-tube and these have to be optimized to get similar values as from the bulk material. Our results studying Zn-3Ag stents in a porcine model corroborate previous *in vitro* and *in vivo* results and indicate that Zn-3Ag stents could serve as potential alternative to PLLA or Mg based stents currently in clinical use. Zn-3Ag is characterized by its substantially higher fracture elongation rate compared with other bioresorbable materials such as PLLA, WE43 and thus may be eligible for various mechanically stable stent designs. In summary, we report for the first time that vascular stents made from Zn-3Ag alloy exhibit excellent mechanical properties, good hemocompatibility, no signs of thrombosis, and excellent vascular healing in conjunction with complete neointimal coverage in porcine iliofemoral arteries. Especially those vascular stents that feature a high resistance to breakage may turn out to improve clinical results of vascular stenting. Our findings should inspire further investigations on Zn-3Ag alloy platforms for bioresorbable stents especially for currently excluded clinical applications such as lesions in side branch regions where standard bioresorbable PLLA and Mg- stents- and scaffolds are contraindicated.

## Supporting information

S1 Fig Mechanical stress-strain curve of extruded Zn-3Ag. (TIF)

S1 Table Binding energies and surface elements in at-% of different stent materials. Surface elements were measured by XPS after sputtering for 60 s, 180 s, and 300 s.
(DOCX)

## References

[1] King SB, Meier B. Interventional treatment of coronary heart disease and peripheral vascular disease. Circulation 2000;102(20 Suppl 4):IV81–6. 11080136 10.1161/01.cir.102.suppl_4.iv-81

[2] Mani G, Feldman MD, Patel D, Agrawal CM. Coronary stents: a materials perspective. Biomaterials 2007;28(9):1689–710. 10.1016/j.biomaterials.2006.11.042 17188349

[3] Moravej M, Mantovani D. Biodegradable metals for cardiovascular stent application: interests and new opportunities, Int. J. Mol. Sci. 2011;12(7):4250–70. 10.3390/ijms12074250 21845076 PMC3155349

[4] Hehrlein C, Weinschenk I, Metz J. Long period of balloon inflation and the implantation of stents potentiate smooth muscle cell death. Possible role of chronic vascular injury in restenosis. Int. J. Cardiovasc. Intervent. 1999;2(1):21–6. 12623383 10.1080/acc.2.1.21.26

[5] Haude M, Ince H, Abizaid A, Toelg R, Lemos PA, von Birgelen C, et al Safety and performance of the second-generation drug-eluting absorbable metal scaffold in patients with de-novo coronary artery lesions (BIOSOLVE-II): 6 month results of a prospective, multicentre, non-randomised, first-in-man trial. Lancet 2016;387(10013): 31–9. 10.1016/S0140-6736(15)00447-X 26470647

[6] Serruys PW, Chevalier B, Sotomi Y, Cequier A, Carrié D, Piek JJ, et al Comparison of an everolimus-eluting bioresorbable scaffold with an everolimus-eluting metallic stent for the treatment of coronary artery stenosis (ABSORB II): a 3 year, randomised, controlled, single-blind, multicentre clinical trial. Lancet 2016;388(10059):2479–91. 10.1016/S0140-6736(16)32050-5 27806897

[7] Hehrlein C, Stintz M, Kinscherf R, Schlösser K, Huttel E, Friedrich L, et al Pure beta-particle-emitting stents inhibit neointima formation in rabbits. Circulation 1996;93(4):641–5. 8640989 10.1161/01.cir.93.4.641

[8] Murphy JG, Schwartz RS, Edwards WD, Camrud AR, Vlietstra RE, Holmes DR Jr. Percutaneous polymeric stents in porcine coronary arteries. Initial experience with polyethylene terephthalate stents. Circulation 1992; 86:1596–04. 1423971 10.1161/01.cir.86.5.1596

[9] Wan P, Wu J, Tan L, Zhang B, Yang K. Research on super-hydrophobic surface of biodegradable magnesium alloys used for vascular stents. Mater. Sci. Eng. C Mater. Biol. Appl. 2013;33(5):2885–90. 10.1016/j.msec.2013.03.017 23623110

[10] Tan LP, Venkatraman SS, Joso JF, Boey FY. Collapse pressures of bilayered biodegradable stents. J. Biomed. Mater. Res. B Appl. Biomater. 2006;79(1):102–7. 10.1002/jbm.b.30518 16544311

[11] Campos CM, Lemos PA. Bioresorbable vascular scaffolds: novel devices, novel interpretations, and novel interventions strategies. Catheter. Cardiovasc. Interv. 2014;84(1):46–7. 10.1002/ccd.25541 24975261

[12] Tenekecioglu E, Farooq V, Bourantas CV, Silva RC, Onuma Y, Yilmaz M, et al Bioresorbable scaffolds: a new paradigm in percutaneous coronary intervention. BMC Cardiovasc. Disord. 2016;16:38–49. 10.1186/s12872-016-0207-5 26868826 PMC4751731

[13] Bornapour M, Muja N, Shum-Tim D, Cerruti M, Pekguleryuz M. Biocompatibility and biodegradability of Mg-Sr alloys: the formation of Sr-substituted hydroxyapatite. Acta Biomater. 2013;9(2):5319–30. 10.1016/j.actbio.2012.07.045 22871640

[14] Di Mario C, Griffiths H, Goktekin O, Peeters N, Verbist J, Bosiers M, et al Drug-eluting bioabsorbable magnesium stent. J. Interv. Cardiol. 2004;17(6):391–5. 10.1111/j.1540-8183.2004.04081.x 15546291

[15] Heublein B, Rohde R, Kaese V, Niemeyer M, Hartung W, Haverich A. Biocorrosion of magnesium alloys: a new principle in cardiovascular implant technology? Heart 2003;89(6):651–6. 12748224 10.1136/heart.89.6.651 PMC1767674

[16] Feyerabend F, Fischer J, Holtz J, Witte F, Willumeit R, Drücker H, et al Evaluation of short-term effects of rare earth and other elements used in magnesium alloys on primary cells and cell lines. Acta Biomater. 2010;6(5):1834–42. 10.1016/j.actbio.2009.09.024 19800429

[17] Zhang S, Zhang X, Zhao C, Li J, Song Y, Xie C, et al Research on an Mg-Zn alloy as a degradable biomaterial. Acta Biomater. 2010;6(2):626–40. 10.1016/j.actbio.2009.06.028 19545650

[18] Shearier ER, Bowen PK, He RJW, Drelich A, Drehlich J, Goldman J, et al In vitro cytotoxicity, adhesion, and proliferation of human vascular cells exposed to zinc. ACS. Biomater. Sci. Eng. 2016;2(4):634–42. 10.1021/acsbiomaterials.6b00035 PMC5102339 27840847

[19] Bowen PK, Shearier ER, Zhao S, Guillory RJ, Zhao F, Goldman J, et al Biodegradable metals for cardiovascular stents: from clinical concerns to recent Zn-Alloys. Adv. Healthc. Mater. 2016;5(10):1121–40. 10.1002/adhm.201501019 27094868 PMC4904226

[20] Konstantinos K, Constantinides G, Georgiou H, Cristea D, Gabor C, Munteanu D, et al Multi-scale mechanical investigation of stainless steel and cobalt–chromium stents. J Mech Behavior Biomed Mat 2016;40:240–51. 10.1016/j.jmbbm.2014.09.010 25255419

[21] Kilkenny C, Browne WJ, Cuthil IC, Emerson M, Altman DG. Improving bioscience research reporting:The ARRIVE Guidelines for Reporting Animal Research. PLoS Biol 2010;8(6):e1000412 10.1371/journal.pbio.1000412 20613859 PMC2893951

[22] Fukada T, Yamasaki S, Nishida K, Murakami M, Hirano T. Zinc homeostasis and signaling in health and diseases: Zinc signaling. J. Biol. Inorg. Chem. 2011;16(7):1123–34. 10.1007/s00775-011-0797-4 21660546 PMC3176402

[23] Saper RB, Rash R. Zinc: an essential micronutrient, Am. Fam. Physician 2009;79(9):768–72. 20141096 PMC2820120

[24] Persaud-Sharma D, Budiansky N, McGoron AJ. Biocompatibility Assessment of Novel Bioresorbable Alloys Mg-Zn-Se and Mg-Zn-Cu for Endovascular Applications: In- Vitro Studies. J. Biomim. Biomater. Tissue Eng. 2013;17:25–44. 10.4028/www.scientific.net/JBBTE.17.25 24058329 PMC3777539

[25] Gong H, Wang K, Strich R, Zhou JG. In vitro biodegradation behavior, mechanical properties, and cytotoxicity of biodegradable Zn-Mg alloy. J. Biomed. Mater. Res. B Appl. Biomater. 2015;103(8):1632–40. 10.1002/jbm.b.33341 25581552 PMC5444388

[26] Niu J, Tang Z, Huang H, Pei J, Zhang H, Yuan G, et al Research on a Zn-Cu alloy as a biodegradable material for potential vascular stents application. Mater. Sci. Eng. C Mater. Biol. Appl. 2016;69:407–13. 10.1016/j.msec.2016.06.082 27612729

[27] Wu Z, Curtin WA. The origins of high hardening and low ductility in magnesium. Nature 2015;526;62–7. 10.1038/nature15364 26390153

[28] Ma J, Zhao N, Zhu D. Bioabsorbable zinc ion induced biphasic cellular responses in vascular smooth muscle cells. Sci Rep. 2016:6:26661 10.1038/srep26661 27248371 PMC4888653

[29] Ma J, Zhao N, Zhu D. Endothelial Cellular Responses to Biodegradable Metal Zinc. ACS Biomater. Sci. Eng. 2015:1(11):1174–82. 10.1021/acsbiomaterials.5b00319 PMC5038919 27689136

[30] Werner M, Micari A, Cioppa A, Vadalà G, Schmidt A, Sievert H, et al Evaluation of the biodegradable peripheral Igaki-Tamai stent in the treatment of de novo lesions in the superficial femoral artery: the GAIA study. JACC Cardiovasc. Interv. 2014;7(3):305–12. 10.1016/j.jcin.2013.09.009 24529932

[31] Kfoury G, Raquez GM, Hassouna F, Odent J, Toniazzo V, Ruch D, et al Recent advances in high performance poly(lactide): from "green" plasticization to super-tough materials via (reactive) compounding. Front Chem 2013;1:32–46. 10.3389/fchem.2013.00032 24790960 PMC3982567

[32] Yang H, Wang C, Liu C, Chen H, Wu Y, Han J, et al Evolution of the degradation mechanism of pure zinc stent in the one-year study of rabbit abdominal aorta. Biomaterials 2017;145:92–105. 10.1016/j.biomaterials.2017.08.022 28858721

[33] Yang H, Qu X, Lin W, Wang C, Zhu D, Dai K, et al *In vitro* and *in vivo* studies on zinc-hydroxyapatite composites as novel biodegradable metal matrix composite for orthopedic applications. Acta Biomater. 2018;71:200–14. 10.1016/j.actbio.2018.03.007 29530820

[34] Zhu D, Su Y, Young ML, Ma J, Zheng Y, Tang L. Biological Responses and Mechanisms of Human Bone Marrow Mesenchymal Stem Cells to Zn and Mg Biomaterials. ACS Appl. Mater. Interfaces, 2017:9(33):27453–61. 10.1021/acsami.7b06654 28787130

[35] Sikora-Jasinska M, Mostaed E, Mostaed A, Beanland R, Mantovani D, Vedani M. Fabrication, mechanical properties and in vitro degradation behavior of newly developed Zn-Ag alloys for degradable implant applications. Mater Sci Eng C Mater Biol Appl 2017;77:1170–81. 10.1016/j.msec.2017.04.023 28531993

