# Supplementary figures and images for: Zn-alloy provides a novel platform for mechanically stable bioresorbable vascular stents
Source: PLoS One. 2019 Jan 2;14(1):e0209111. doi: 10.1371/journal.pone.0209111 (PMC6314592; doi:10.1371/journal.pone.0209111)

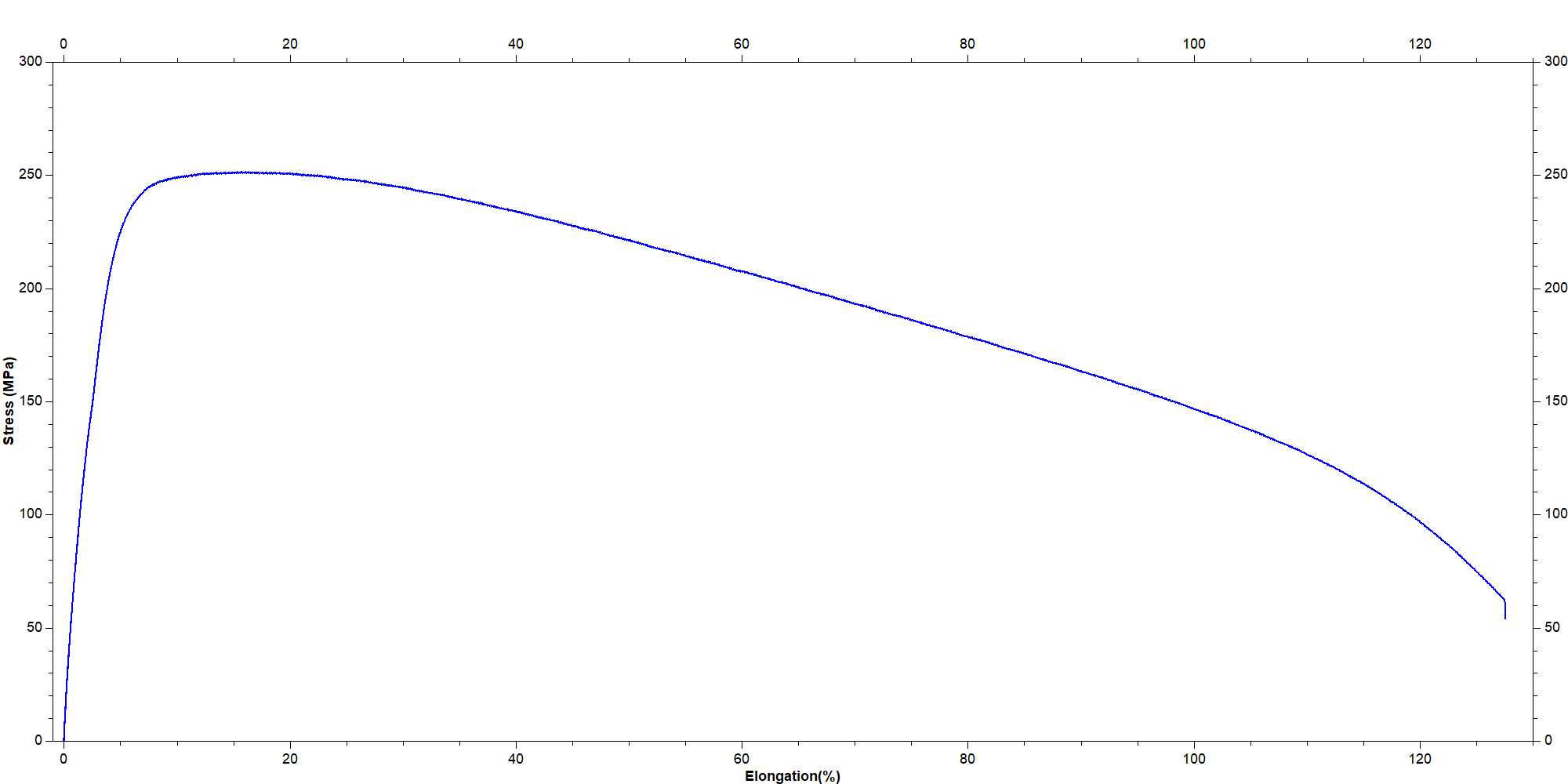

Supplement: S1 Fig — (TIF) [file pone.0209111.s001.tif]
