# Supplementary material for: Zn-alloy provides a novel platform for mechanically stable bioresorbable vascular stents
Source: PLoS One. 2019 Jan 2;14(1):e0209111. doi: 10.1371/journal.pone.0209111 (PMC6314592; doi:10.1371/journal.pone.0209111)
Supplement: S1 Table — Surface elements were measured by XPS after sputtering for 60 s, 180 s, and 300 s. (DOCX) [file pone.0209111.s002.docx]

Binding energies and element concentration in at-% of different stent materials

| **Sample** | **Metal** | **BE** | **O 1s** | **BE** | **N 1s** | **BE** | **Ca 2p** | **BE** | **C 1s** | **BE** | **P 2s** | **BE** | **N a 1s** | **BE** | **M_x_O_y_N_z_Ca_u_C**_v_**P_w_** |
| --- | --- | --- | --- | --- | --- | --- | --- | --- | --- | --- | --- | --- | --- | --- | --- |
|  | **at-%** | **[eV]** | **at-%** | **[eV]** | **at-%** | **[eV]** | **at-%** | **[eV]** | **At-%** | **[eV]** | **at-%** | **[eV]** | **at-%** | **[eV]** |  |
| **Nitinol** | Ti 2p | |  |  |  |  |  |  |  |  |  |  |  |  |  |
| surface | 1.31 | 457.68 | 38.37 | 530.55 | 7.88 | 399.05 | - | - | 47.48 | 284.92 | 4.97 | 189.90 | - | - |  |
| 6 nm | 3.05 | 457.12 | 47.71 | 530.12 | 5.97 | 399.36 | 1.72 | 347.72 | 39.56 | 284.66 | - | - | 1.98 | 1070.23 | TiO_15.5_N_2_Ca_0.56_C_13_Na_0.64_ |
| 18 nm | 6.77 | 457.61 | 65.04 | 531.20 | 5.19  1.06 | 405.98  399.52 | - | - | 23.93 | 285.18 | - | - | - | - | TiO_9.6_N_0.6_C_3.5_ |
| 30 nm | 8.16 | 456.76 | 59.81 | 530.26 | 9.22  4.46 | 405.76  399.15 | - | - | 18.35 | 284.33 | - | - | - | - | TiO_7.3_N_1.1_C_2.2_ |
| **WE 43** | Mg |  |  |  |  |  |  | Ca 2s |  |  |  | P 2p |  |  |  |
| surface | 20.91 | 1304.56 | 54.41 | 532.43 | - | - | 6.36 | 440.05 | 6.19 | 285.02 | 12.14 | 134.20 | - | - |  |
| 6 nm | 20.97 | 1303.58 | 55.47 | 531.30 | - | - | 4.20 | 439.13 | 7.97 | 284.82 | 11.40 | 133.57 | - | - | MgO_2.6_Ca_0.2_C_0.38_P_0.54_ |
| 18 nm | 18.48 | 1304.38 | 54.30 | 532.41 | - | - | 4.47 | 440.75 | 13.84 | 285.07 | 8.91 | 134.45 | - | - | MgO_2.9_Ca_0.24_C_0.75_P_0.48_ |
| 30 nm | 23.80 | 1304.45 | 59.62 | 532.27 | - | - | 5.13 | 440.09 | - | - | 11.45 | 134.56 | - | - | MgO_2.5_Ca_0.2_P_0.48_ |
| **Zn** | Zn |  |  |  |  |  |  |  |  |  |  |  |  |  |  |
| surface | 3.75 | 1021.62 | 20.46 | 530.86 | 11.86 | 398.79 | - | - | 63.93 | 284.60 | - | - | - | - |  |
| 6 nm | 11.20 | 1023.32 | 15.78 | 532.47 | 10.83 | 399.01 | - | - | 62.17 | 284.47 | - | - | - | - | ZnO_1.4_N_0.68_C_5.55_ |
| 18 nm | 13.01 | 1024.23 | 16.06 | 533.52 | 9.45 | 399.86 | - | - | 61.48 | 285.46 | - | - | - | - | ZnO_1.2_N_0.7_C_4.7_ |
| 30 nm | 11.29 | 1023.00 | 13.79 | 532.27 | 9.31 | 398.84 | - | - | 65.61 | 284.47 | - | - | - | - | ZnO_1.2_N_0.8_C_5.8_ |
| **Zn-3Ag** | Zn |  |  |  |  |  |  |  |  |  |  |  |  |  |  |
| surface | 4.24 | 1020.54 | 27.33 | 530.33 | 10.94 | 400.21 | - | - | 52.00 | 284.22 |  |  | - | - |  |
| 6 nm | 12.31 | 1021.77 | 35.93 | 531.46 | 4.76 | 399.36 | - | - | 34.54 | 285.00 | 11.25 | 190.11 | 1.21 | 1071.09 | ZnO_2.9_N_0.39_C_2.8_P_0.9_Na_0.1_ |
| 18 nm | 17.71 | 1021.00 | 49.45 | 530.58 | 3.68 | 397.14 | - | - | 20.30 | 284.42 | 8.11 | 189.35 | 0.72 | 1070.78 | ZnO_2.8_N_0.1_C_1.1_P_0.8_Na_0.04_ |
| 30 nm | 24.70 | 1021.93 | 64.22 | 531.55 | 14.05  5.58 | 399.07  406.47 | - | - | - | - | - | - | - | - | ZnO_2.6_N_0.45_ |
| **PLLA** |  |  |  |  |  |  |  |  |  |  |  |  |  |  |  |
| surface | - | - | 26.37 | 531.89 | - | - | - | - | 73.63 | 285.02 | - | - | - | - |  |
| 15 nm | - | - | 27,.53 | 532.44 | - | - | 5.53 | 348.94 | 66.94 | 284.94 | - | - | - | - | C_12_O_5_Ca |
| 50 nm | - | - | 25.47 | 532.61 | - | - | 5.77 | 349.34 | 68.76 | 284.70 | - | - | - | - | C_12_O_4.4_ca |
| 80 nm | - | - | 26.95 | 532.62 | - | - | 4.20 | 348.83 | 68.84 | 284.71 | - | - | - | - | C_16.4_O_6.4_Ca |
